# Sieving Hydrogen Isotopes via Machine Learning Assisted Chemical Vapor Deposition (CVD) of High‐Quality Monolayer Hexagonal Boron Nitride (h‐BN) on Iron Foils

**DOI:** 10.1002/adma.202511868

**Published:** 2025-11-28

**Authors:** Pavan Chaturvedi, Andrew E. Naclerio, Saban M. Hus, Ivan V. Vlassiouk, Nickolay Lavrik, Marti Checa, Liam Collins, An‐Ping Li, Piran R. Kidambi

**Affiliations:** ^1^ Chemical and Biomolecular Engineering Department Vanderbilt University Nashville TN 37212 USA; ^2^ Center for Nanophase Materials Sciences Oak Ridge National Laboratory Oak Ridge TN 37831 USA; ^3^ Mechanical and Aerospace Engineering Department University of Florida Gainesville FL 32611 USA

**Keywords:** atomically thin membranes, ceramic membranes, chemical vapor deposition (CVD), hexagonal boron nitride (h‐BN), iron, isotope separation, proton‐deuteron (H^+^/D^+^) separation, sieving hydrogen isotopes, sub‐atomic separations

## Abstract

Atomically thin two‐dimensional (2D) ceramics, such as monolayer hexagonal boron nitride (h‐BN), present potential for disruptive advances in separations. However, sub‐atomic scale separation of hydrogen isotopes (H^+^/D^+^) require near pristine 2D material membranes, and scalable synthesis of such high‐quality h‐BN comparable to mechanically exfoliated crystals remains a significant challenge. Here, we report a scalable Fe‐catalyzed chemical vapor deposition (CVD) process for bottom‐up synthesis of large‐area, high‐quality monolayer h‐BN films, overcoming key limitations of conventional ammonia‐based routes. By leveraging mechanistic insights and higher CVD temperatures, we suppress multilayer formation and achieve uniform monolayer h‐BN coverage on commercially available Fe foils. Machine learning enables systematic exploration of the complex, multi‐dimensional CVD parameter space (growth time, temperature, precursor temperature, multilayer faction, coverage), providing data‐driven approaches to visualize and identify process regimes facilitating predominantly monolayer h‐BN growth with minimal secondary nuclei/ad‐layers. The optimized Fe‐catalyzed CVD *h*‐BN membranes show high‐quality as observed by proton/deuteron (H^+^/D^+^) selectivity ≈8.45, approaching the highest quality benchmark of mechanically exfoliated h‐BN (H^+^/D^+^ selectivity ≈10) as well as significantly outperforming Cu‐catalyzed CVD *h*‐BN membranes (H^+^/D^+^ selectivity ≈3.62, control selectivity ≈1.7). Our work provides a scalable cost‐effective route for high‐quality monolayer h‐BN synthesis for sub‐atomic scale separations (H^+^/D^+^) and demonstrates the broader potential of machine learning‐guided optimization of CVD for advancing synthesis of 2D materials.

## Graphical abstract

Sub‐atomic scale separation of hydrogen isotopes (H^+^/D^+^) require near pristine h‐BN membranes, and scalable synthesis of such high‐quality h‐BN comparable to mechanically exfoliated crystals remains a significant challenge. This study reports a scalable Fe‐catalyzed Machine‐Learning Enabled Chemical vapor deposition process for bottom‐up large‐area, high‐quality monolayer h‐BN film synthesis and demonstrate H^+^/D^+^ separation with selectivity approaching mechanically exfoliated h‐BN.

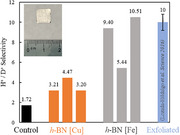

## 1 Introduction

Atomically thin (2D) materials such as monolayer graphene and hexagonal boron nitride (*h*‐BN) present fundamentally new opportunities for separation processes,^[^ ^1^, ^2^, ^3^, ^4^ ^]^ including ionic and molecular sieving,^[^ ^5^, ^6^, ^7^ ^]^ dialysis,^[^ ^8^, ^9^, ^10^, ^11^, ^12^, ^13^ ^]^ gas separations,^[^ ^14^, ^15^, ^16^ ^]^ proton exchange membranes,^[^ ^17^, ^18^, ^19^, ^20^, ^21^ ^]^ as well as sub‐atomic separations of hydrogen isotopes (H^+^/D^+^/T^+^) with orders of magnitude lower energy requirements than conventional separation processes.^[^ ^1^, ^2^, ^3^, ^4^, ^5^, ^6^, ^7^, ^22^, ^23^, ^24^, ^25^, ^26^ ^]^ Monolayer h‐BN's unique combination of atomic thinness, exceptional chemical and thermal stability,^[^ ^27^ ^]^ and ability to sustain lattice defects that manifest as nanopores presents potential for creating atomically thin ceramic membranes^[^ ^5^ ^]^ with high permeance (atomic‐thinness) and high selectivity (size‐selective pores).^[^ ^1^, ^5^, ^6^, ^7^, ^28^, ^29^, ^30^ ^]^ In addition to atomically‐thin membranes for separations,^[^ ^1^, ^4^, ^6^, ^31^, ^32^ ^]^ monolayer h‐BN is also desirable for applications such as oxidation protection,^[^ ^33^, ^34^, ^35^, ^36^ ^]^ tunnel transistors,^[^ ^37^, ^38^ ^]^ spin tunnel junctions,^[^ ^39^, ^40^, ^41^ ^]^ optics,^[^ ^42^, ^43^, ^44^ ^]^ among others.^[^ ^27^, ^43^, ^45^, ^46^ ^]^

Sub‐atomic scale separations of hydrogen isotopes (attributed to differences in vibrational zero‐point energy of the incident protons and deuterons on the 2D crystals)^[^ ^3^, ^4^ ^]^ impose extremely stringent material requirements on monolayer h‐BN quality than most other applications.^[^ ^27^ ^]^ For example, point defects or lattice vacancies could create non‐selective leakage pathways, drastically undermining atomically thin membrane performance and selectivity.^[^ ^2^, ^4^, ^6^, ^7^, ^17^, ^18^, ^19^, ^20^, ^21^ ^]^ Hydrogen isotope separation (distinguishing between H⁺ and D⁺) requires extremely high‐quality, monolayer h‐BN membranes with minimal defects comparable to mechanically exfoliated crystals.^[^ ^1^, ^2^, ^3^, ^4^, ^27^ ^]^ However, high‐quality h‐BN synthesis has largely remained limited to high pressure and temperature processes developed to produce bulk crystal which are then exfoliated mechanically to produce monolayer flakes.^[^ ^27^, ^47^ ^]^ Recent advances in metal flux methods have also enabled synthesis of bulk h‐BN crystals of high quality,^[^ ^46^, ^47^, ^48^, ^49^, ^50^ ^]^ but the isolation and fabrication of large‐area monolayers from bulk crystals remains a significant challenge.^[^ ^27^ ^]^

In this context, chemical vapor deposition (CVD) using boron and nitrogen‐containing precursors on catalytic metal substrates (Cu, Ni, Co, Pt, Au, and other alloys foils or films) has emerged as the preferred route for cost‐effective, scalable, bottom‐up synthesis of large‐area monolayer h‐BN films.^[^ ^5^, ^41^, ^51^, ^52^, ^53^, ^54^, ^55^, ^56^, ^57^, ^58^ ^]^

Leveraging initial reports of graphene CVD on Cu foil,^[^ ^59^ ^]^ h‐BN synthesis on Cu foil was demonstrated^[^ ^58^ ^]^ and remained the most well studied in literature^[^ ^27^, ^60^ ^]^ with approaches extending it to single‐crystalline Cu(110), Cu(111) as well as Au to achieve higher quality h‐BN monolayer films.^[^ ^56^, ^61^, ^62^ ^]^ However, intrinsic defects are un‐avoidable in bottom‐up synthesis of 2D materials via CVD^[^ ^27^, ^63^ ^]^ and the lack of facile quantitative characterization techniques (such as Raman for graphene)^[^ ^64^ ^]^ limits progress in synthesis of high‐quality monolayer *h‐*BN.^[^ ^5^ ^]^

Iron (Fe) catalysts have been shown to enable the growth of large monolayer h‐BN domains.^[^ ^65^ ^]^ However, the high solubility of boron and nitrogen in the Fe catalyst bulk (≈39 ppm B and ≈250 ppm N at 1050 °C) inevitably leads to the formation of multilayers during growth (isothermal) or cooling (precipitation) and stabilizing uniform monolayer h‐BN remains challenging.^[^ ^54^, ^66^, ^67^ ^]^ While approaches such as ammonia pre‐treatment to fill the Fe bulk reservoir with N have been explored to mitigate bulk uptake of elemental species and promote monolayer h‐BN growth,^[^ ^54^ ^]^ the toxicity/corrosivity of ammonia necessitates not only additional safety measures and specialized equipment, but excess ammonia exposure can lead to formation of iron nitrides detrimental to h‐BN growth.^[^ ^68^, ^69^ ^]^

Here, we present a facile and novel approach for monolayer *h*‐BN synthesis via CVD by increasing the growth temperatures to enhance solubility of boron and nitrogen in the Fe catalyst, driving them deeper into the catalyst bulk. This reduces supersaturation near the surface, thereby suppressing multilayer formation. To optimize the growth process, we employ machine learning to systematically explore the multi‐dimensional CVD parameter space and identify the conditions that maximize monolayer coverage while minimizing secondary nucleation of multi‐layers/ad‐layers. We benchmark the quality of the synthesized *h‐*BN monolayer via fabrication of membranes for H^+^/D^+^ separations, achieving an H⁺/D⁺ selectivity (≈8.45) comparable to the highest‐quality mechanically exfoliated h‐BN (≈10) and surpassing quality of CVD h‐BN grown on copper foils (≈3.62). To the best of our knowledge this is the highest H⁺/D⁺ selectivity reported for CVD grown h‐BN. These results establish a scalable pathway for synthesizing high‐quality monolayer h‐BN membranes suitable for extremely demanding sub‐atomic separations that has remained elusive.

## 2 Results and Discussion

### 2.1 Large‐Area h‐BN Synthesis via Chemical Vapor Deposition (CVD) on Fe Foil

**Figure 1:**
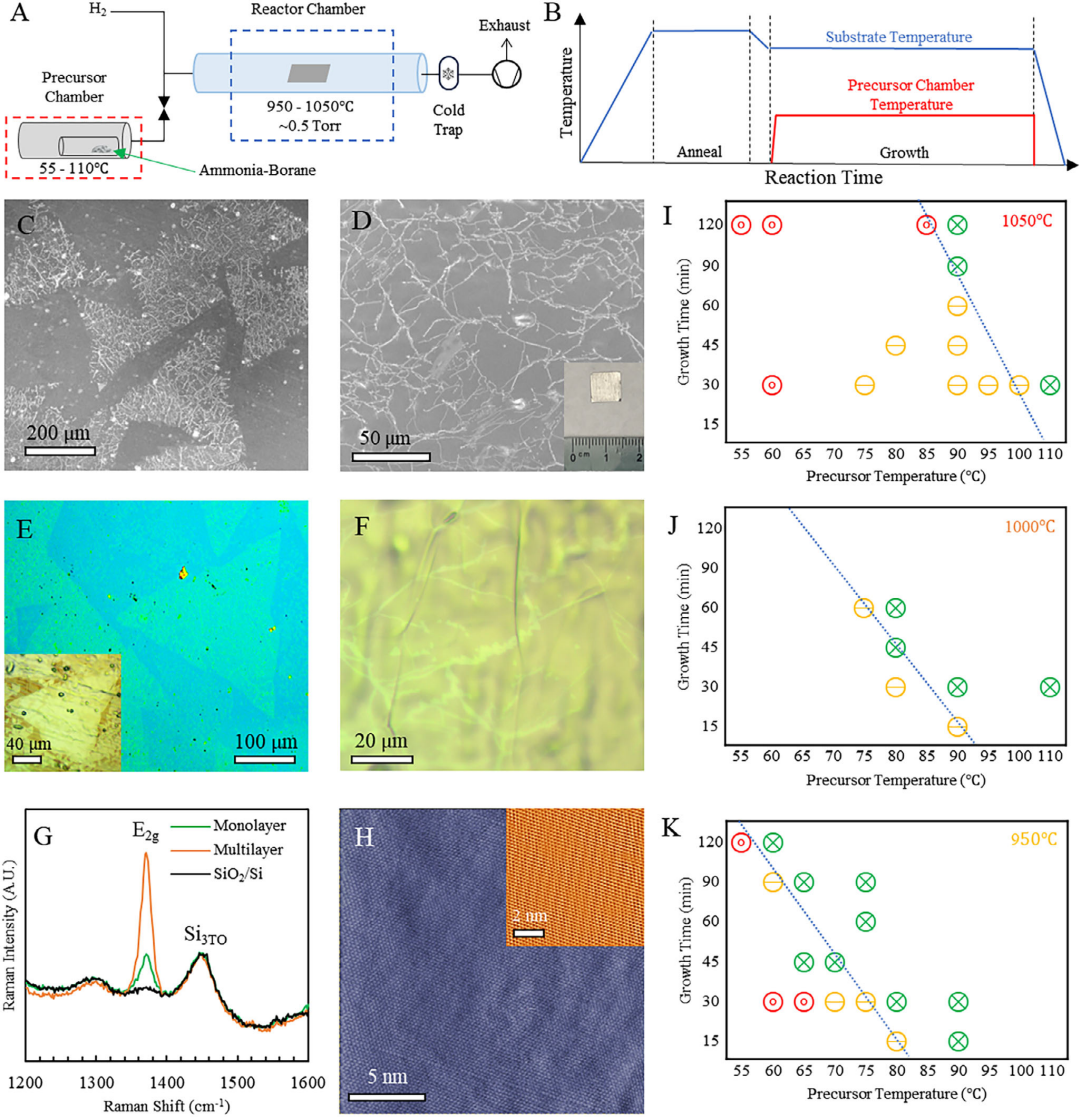
*h*‐BN synthesis via chemical vapor deposition (CVD) on Fe foil. A) Schematic of the low‐pressure CVD reactor using ammonia‐borane powder as precursor. B) CVD reactor temperature profiles. After annealing the Fe foil in H_2_, ammonia‐borane is heated in the side chamber to deliver precursor to the CVD reactor. SEM images of C) un‐merged triangular *h‐*BN domains and D) continuous *h‐*BN film on Fe foil (≈127 µm thick). Wrinkles in the triangular domains as well as the continuous films indicate the presence of *h*‐BN on Fe foil. Inset in D shows centimeter‐scale areas over which h‐BN is synthesized via CVD. E) Optical image of *h*‐BN domains transferred to 300 nm SiO_2_/Si wafer. Inset shows optical image of un‐merged triangular *h‐*BN domains on Fe foil after heating in air at ≈200 °C, for ≈1–3 min. F) Optical image of continuous *h‐*BN film on Fe foil after heating in air. Multilayer regions in the films are visible, specifically underneath wrinkles. G) Raman spectra acquired on monolayer and multilayer regions of the *h*‐BN film transferred to 300 nm SiO_2_/Si wafer as well as the bare 300 nm SiO_2_/Si wafer as a control. The peak ≈1372 cm^−1^ is consistent with the E_2g_ phonon mode of *h‐*BN. H) Atomic resolution scanning tunnelling microscopy image (STM, collected at −1.8 V, 100 pA over 20 × 20 nm area) of *h‐*BN on Fe foil. Inset shows FFT filtered STM image of a 9 × 9 nm region. *h*‐BN film coverage on the Fe foil as function of precursor temperature and growth time at growth temperatures of I) 1050 °C, J) 1000 °C and K) 950 °C. Green points indicate a complete *h*‐BN film was grown, yellow points indicate incomplete *h‐*BN film growth, and red indicates no *h*‐BN grown. Blue dotted line is a guide for the eye indicating regions of transition from incomplete to complete *h*‐BN film coverage. Note the blue line shifts to the right with increasing temperatures. Also see Figure S1 (Supporting Information) for UV–vis absorption spectra and AFM of the *h‐*BN domains/films.

*h‐*BN is synthesized in a custom‐built hot‐walled tube furnace reactor^[^ ^5^, ^70^ ^]^ (**Figure** **1**A) via low‐pressure chemical vapor deposition (LPCVD) using iron (Fe) foil as the catalytic growth substate after pre‐treatment and annealing in H_2_ (see Experimental Section). Ammonia‐borane powder is used as the precursor (stochiometric ratio of B:N = 1) for h‐BN synthesis and heated in a side chamber and introduced into the CVD reactor in a background of H_2_ gas for the desired growth time (Figure 1B). The Fe foil dissociates the precursor and catalyzes *h*‐BN formation via nucleation of domains (Figure 1C,E) that grow with time and eventually merge to form a continuous polycrystalline h‐BN film (Figure 1D,F). The triangular shape of the h‐BN domains (Figure 1C,E) is attributed to the higher stability of N‐terminated crystal edges over B‐terminated edges^[^ ^27^, ^41^, ^51^, ^55^, ^71^ ^]^ and optical images acquired after oxidation of the Fe foil by heating in air (see Experimental Section, inset in Figure 1E,F) as well as after transfer to 300 nm SiO_2_/Si wafer (Figure 1E) confirm the presence of triangular domains consistent with SEM images. Atomic force microscopy (AFM) of the transferred triangular domains shows a thickness of ≈0.45 nm, consistent with monolayer *h*‐BN (Figure S1B,C, Supporting Information).^[^ ^27^, ^41^, ^55^, ^58^, ^71^ ^]^

The line like features in the SEM images (Figure 1C,D) within the triangular domains as well as the continuous films are wrinkles formed due to differences in thermal expansion between the Fe foil and monolayer h‐BN. Additionally, multilayer regions (brighter regions) are visible in SEM (Figure 1D) and optical images (Figure 1F) of complete films, specifically underneath wrinkles. Raman spectra (Figure 1G) acquired on the monolayer and multilayer regions of the *h*‐BN film transferred to 300 nm SiO_2_/Si wafer shows a peak ≈1372 cm^−1^ consistent with the E_2g_ phonon mode of *h‐*BN (Figure 1G).^[^ ^72^ ^]^ The full width half‐maximum (FWHM) of <20 cm^−1^ (≈18.5 cm^−1^ for Fe‐hBN) for the peak ≈1372 cm^−1^ indicates high quality of the synthesized h‐BN.^[^ ^72^ ^]^ UV‐vis absorption spectra acquired on continuous *h‐*BN films transferred to fused quartz (Figure S1A, Supporting Information) show an optical band gap E_g_ ≈5.9 eV (computed via a Tauc plot, see inset of Figure S1A, Supporting Information) consistent with high quality *h*‐BN.^[^ ^73^, ^74^ ^]^ Atomic resolution scanning tunnelling microscopy (STM) of h‐BN on Fe foil further confirms the presence of a hexagonal honey‐comb lattice indicating high crystalline quality (Figure 1H).

### 2.2 Leveraging Mechanistic Insights and Machine Learning for CVD of High‐Quality Monolayer h‐BN

Having confirmed synthesis of h‐BN via CVD on Fe foil, *i)* we proceed to systematically explore the multi‐dimensional CVD parameter space (growth time, temperature, precursor temperature, multilayer faction, coverage), *ii)* obtain mechanistic understanding of h‐BN growth, and *iii)* leverage machine learning methods to identify regimes that yield the high‐quality monolayer *h*‐BN, with minimal multilayers on Fe foil.

For a systematic data‐driven machine learning approach, we initially study *h*‐BN film coverage on the Fe foil as function of precursor temperature, growth time, and growth temperature (Figure 1I–K). SEM images of the catalyst surface after growth are analyzed using contrast thresholding (see Figure 3A) to extract *h*‐BN surface coverage. In Figure 1I–K, the green points indicate complete *h*‐BN film growth, yellow points indicate incomplete *h‐*BN film growth, and red indicates no *h*‐BN grown and the blue dotted line (guide for the eye) indicates regions of transition from incomplete to complete *h*‐BN films on Fe foil. For a given growth temperature (≈950–1050 °C), *h*‐BN coverage increases with growth times (≈15–120 min, see red‐yellow‐green transitions in the vertical direction), indicating increasing domains size or additional nucleation with time yields higher coverage (Figure 1I–K). Additionally, for a given growth temperature and growth time, *h*‐BN coverage increases with increasing precursor temperature (≈55–110 °C, red‐yellow‐green transitions in the horizontal direction), indicating higher precursor flux results in increased h‐BN surface coverage (Figure 1I–K).^[^ ^75^ ^]^ Notably, with increasing growth temperatures (≈950‐1050 °C) higher precursor flux and longer growth times are necessary to form a complete *h*‐BN film (see shift to the right of the blue dotted line with increasing growth temperature). Increasing growth temperature increases reaction rates (Arrhenius relationship), i.e., precursor decomposition, surface mobility of intermediate species and h‐BN formation rates, however, it also increases desorption from the Fe surface, etching of h‐BN via H_2_ (or trace O_2_ or H_2_O) and solubility for B and N into the Fe bulk.^[^ ^76^, ^77^ ^]^

**Figure 2:**
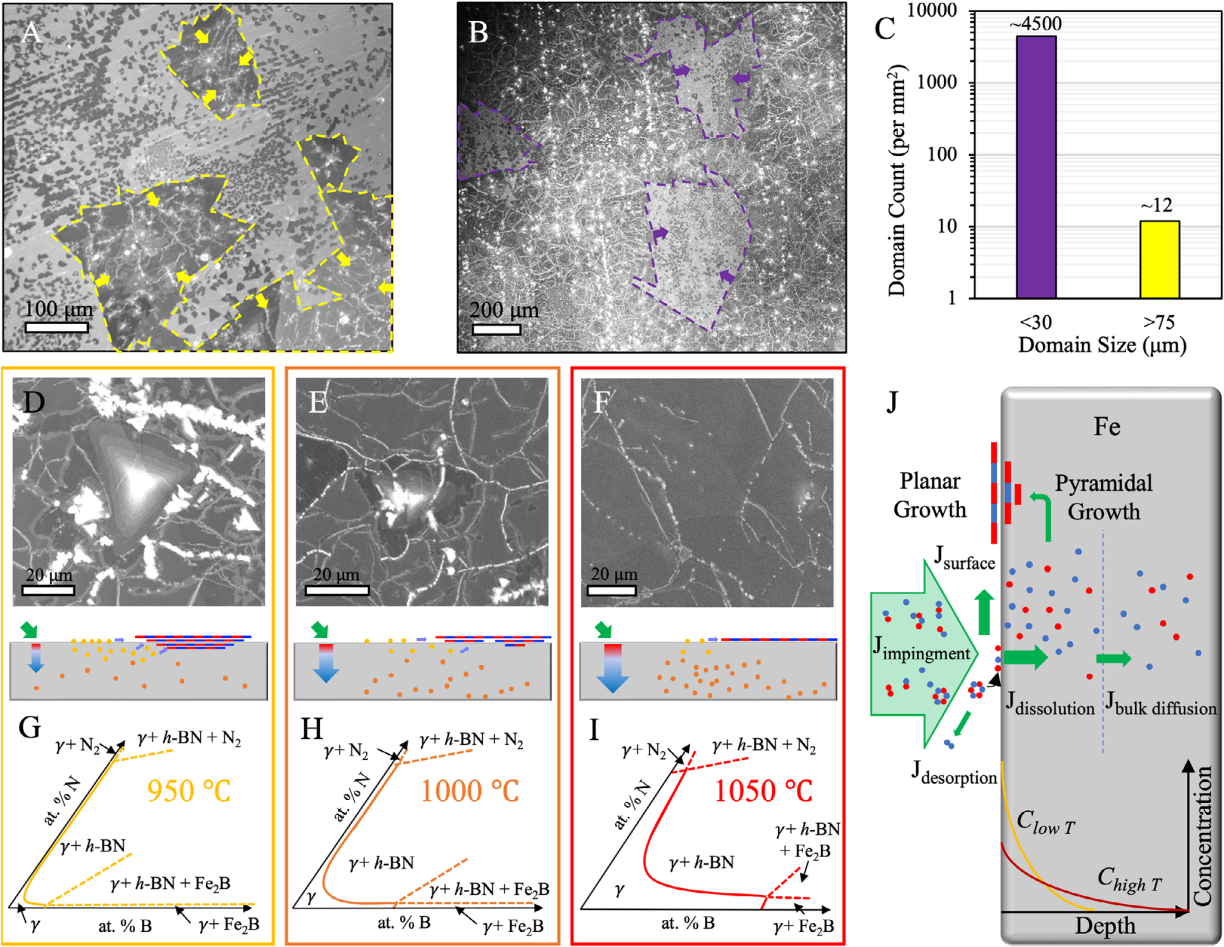
Leveraging CVD growth mechanisms to stabilize monolayer *h*‐BN film on Fe foil. A) SEM image of un‐merged *h*‐BN domains grown on Fe foil (≈127 µm thick). The larger *h‐*BN domains have different shapes compared to the smaller triangular domains. B) Low magnification SEM image of nearly complete *h*‐BN film on Fe foil. Outlined areas are un‐covered Fe regions and contain small triangular h‐BN domains within them. These smaller triangular h‐BN domains form via B and N precipitation induced upon cooling. C) Domain count as a function of domain size for the region seen in A. SEM images of h‐BN films grown at different temperatures D) 950 °C, E) 1000 °C, and F) 1050 °C, respectively. Higher growth temperatures show fewer multilayer domains and schematics below depict precursor flux on the Fe surface and fraction that stays on the surface or in the vicinity of Fe surface, as well as the fraction that diffuses deeper into the Fe bulk. Corresponding Fe‐B‐N phase diagrams for G) 950 °C, H) 1000 °C, and I) 1050 °C, respectively. The phase diagrams indicate increased B and N solubility with increasing temperature before the region for formation of *h*‐BN and other boron compounds is reached. Adapted/re‐drawn from ref. [77] for illustrative purposes with permission of the Minerals, Metals & Materials Society. J) Schematic of proposed model for *h*‐BN growth on Fe foil. Green arrows indicate B and N fluxes in the system. The isothermal formation of monolayer h‐BN and subsequent growth of multilayer pyramidal domains is dependent on the amount of B and N available on or near the Fe catalyst surface. Bulk diffusion and solubility of B and N into the Fe catalyst bulk increases with increased *h*‐BN growth temperature.

**Figure 3:**
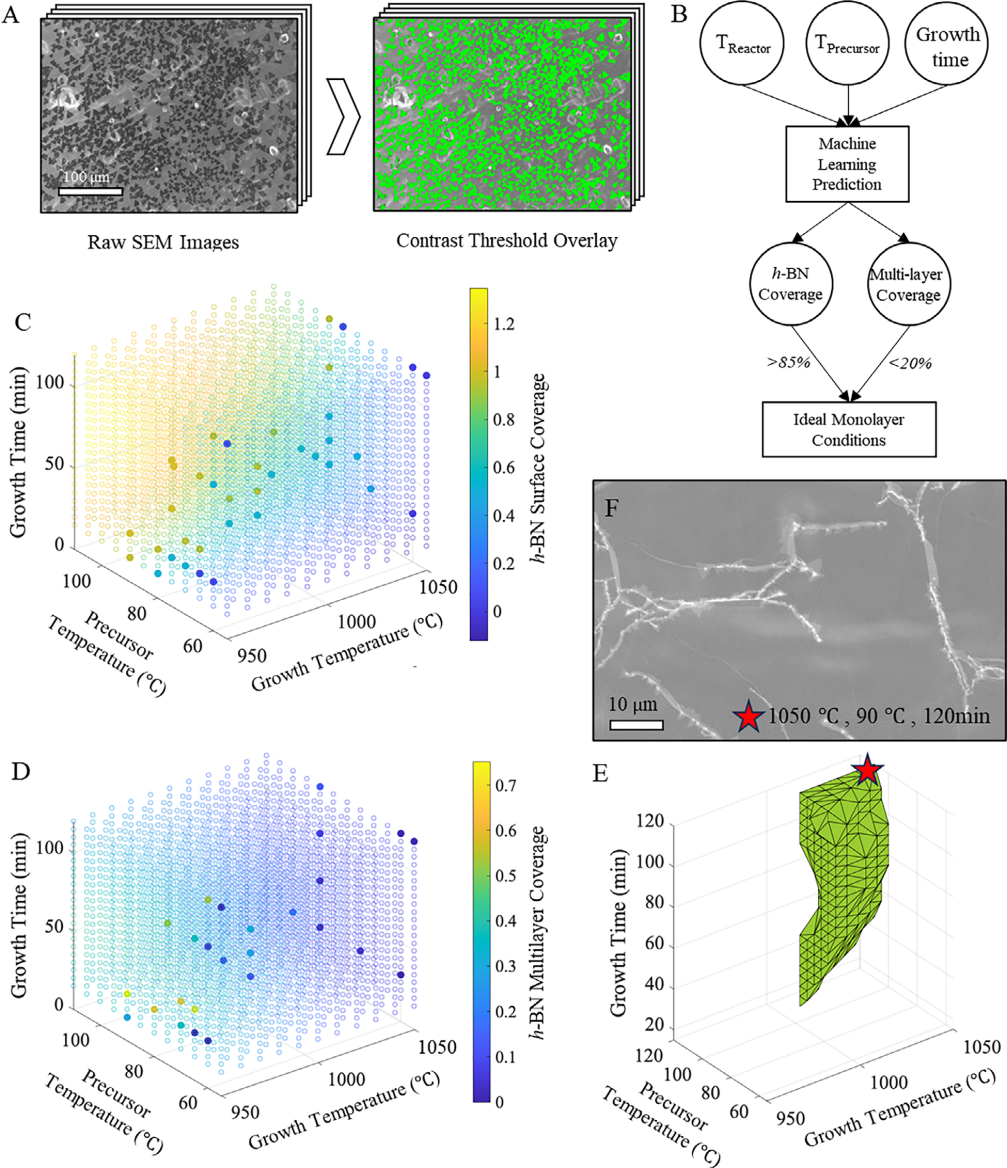
Machine‐learning optimization of CVD parameter space for monolayer h‐BN synthesis on Fe foil. A) Representative contrast thresholding in ImageJ software used to quantify *h*‐BN surface coverage. *h‐*BN surface coverage is binned into no coverage observed (0.0), partial coverage (0.5) and complete film (1.0). Multilayer coverage fraction is computed via contrast thresholding due to the higher brightness in the SEM images. B) Schematic of machine‐learning workflow using the reactor temperature (T _Reactor_), precursor temperature (T _Precursor_), and growth time to predict *h‐*BN coverage and multilayer coverage using a Gaussian regression (see Experimental Section). *h*‐BN coverage >85% and multilayer coverage <20% were used as criteria to identify the parameter space. C) Training data (filled points) and model predicted *h‐*BN surface coverage (empty points) across the parameter space. C) Training data (filled points) and model predicted multilayer coverage (empty points) across the parameter space. E) Visualization of multi‐dimensional CVD parameter space identified via the machine learning model as conducive to monolayer *h*‐BN growth. Also see Figure S2 (Supporting Information). F) SEM of continuous *h‐*BN monolayer film on Fe foil synthesized using machine learning predicted conditions (star symbol), i.e., growth temperature ≈1050 °C, ammonia‐borane precursor temperature ≈90 °C, and growth time ≈120 min, indeed shows predominantly monolayer *h‐*BN with minimal multi‐layer coverage.

Interestingly, for growth runs that yield partial coverage (yellow points in Figure 1I‐K), we observe two distinctly different sizes of h‐BN domains on Fe foil (**Figure** **2**A‐C), i.e., large triangular and irregularly shaped (merged triangles) domains >75µm covering most of the Fe surface and smaller triangular domains (<30 µm) in the remaining area.^[^ ^65^ ^]^ Image analysis of these domains yields a bimodal distribution with ≈4500 domains <30 µm per mm^2^, ≈12 domains >75 µm, but no domains observed between 30 and 75 µm (Figure 2C). Furthermore, the larger *h‐*BN domains seemed influenced by changes in growth time and precursor flux, while the size of smaller domains (<30 µm) were not. The smaller domains morphology, density and rotational alignment shows Fe crystal facet dependence (**Figure** **3**A).^[^ ^78^ ^]^ These observations indicate the larger irregular domains (often containing pyramidal multilayer regions Figure 2D,E) grow isothermally, while the observed smaller domains are formed from B and N precipitation upon cooling,^[^ ^54^, ^65^ ^]^, i.e., domains growing isothermally are significantly larger since they have more time to grow and are consistently supplied with precursor whereas the domains growing via precipitation are smaller due to much shorter growth times and precursor supply is limited by the amount of dissolved B and N in the bulk that can precipitate upon cooling before the temperature drops below the requirement for sufficient surface mobility/attachment of growth species. The coverage of multilayer pyramidal regions under the complete isothermally grown films shows an inverse dependence on h‐BN growth temperature (Figure 2D–F), i.e., lower growth temperature ≈950 °C showed the highest amount of multilayers, while the ≈1050 °C showed the least amount and ≈1000 °C showed an intermediate.^[^ ^54^, ^65^ ^]^ Notably, for growth temperature ≈1050 °C we only observed multilayer pyramidal structures when the precursor flux supplied was in excess.

We rationalize these observations in context of the Fe‐B‐N phase diagrams^[^ ^77^ ^]^ for different growth temperature (Figure 2G–I). The B and N solubility is increased by increasing the CVD temperature before *h*‐BN or borides phases appear, suggesting a larger reservoir for B and N uptake into the Fe bulk. However, despite a larger reservoir for B and N with increased temperature, less multilayers are observed, even for the same extent of coverage compared to growth at lower temperature. Hence, we suggest that at higher temperatures (≈1050 °C), B and N dissolution and diffusion into the larger Fe catalyst bulk reservoir depletes the surface concentration minimizing isothermal multilayer h‐BN growth (Figure 2G–J). Conversely at lower growth temperatures ≈950 °C, lower B and N dissolution and diffusion into a relatively smaller Fe catalyst bulk reservoir results in a higher surface concentration leading to increased pyramidal multilayers forming isothermally (Figure 2G–J). Taken together, the following growth model (Figure 2J) emerges wherein h‐BN growth via CVD on Fe foil is governed by a balance of fluxes of B and N (green arrows). The precursor impinging on the catalyst surface (*J*_impingement_) can dissociate and *i*) desorb from the surface (*J*_desorption_) and exit the CVD system, *ii*) remain on the surface (*J*_surface_), increasing supersaturation for h‐BN nucleation and grow *h*‐BN, and *iii*) dissolve into the Fe catalyst (*J*_dissolution_) and diffuse into the catalyst bulk (*J*_bulk diffusion_). Hence, the isothermal formation of monolayer and subsequent growth of multilayer pyramidal domains is dependent on the amount of B and N available on or near the Fe catalyst surface.^[^ ^54^ ^]^ Bulk diffusion and solubility of B and N into the Fe catalyst bulk increases with increased *h*‐BN growth temperature.^[^ ^77^ ^]^

Given the multi‐dimensional parameter space for *h*‐BN growth on Fe foil, we leverage a machine learning approach to identify regimes in the parameter space conducive to high quality monolayer h‐BN film with minimal multi‐layers (Figure 3; Figure S2, Supporting Information). The SEM images collected over the parameter space (Figure 1 and 2) for reactor/growth temperature, precursor temperature, growth time and corresponding surface coverage and multilayer coverage are contrast thresholded in ImageJ (Figure 3A) and used in a Gaussian process prediction model (Figure 3A,B).^[^ ^60^ ^]^ Using reactor/growth temperature, precursor temperature, growth time as input parameters (3 input parameters), we predict expected *h*‐BN surface coverage and the expected multilayer coverage (2 output parameters) across the parameter space by using the Matérn 5/2 kernel function with separate length scales for each predictor dimension (*m*) (i.e., furnace temperature range, precursor temperature range and growth time).^[^ ^79^ ^]^

The Matérn 5/2 kernel function is defined as:

(1) $$k\left(x_{i},x_{j}|\theta \right)=\sigma_{f}^{2}\left(1+\sqrt{5r}+\frac{5}{3}r^{2}\right)exp\left(-\sqrt{5r}\right)$$

(2) $$r=\sqrt{\sum_{m=1}^{d}\frac{{\left(x_{im}-x_{jm}\right)}^{2}}{\sigma_{m}^{2}}}$$

where σ_*f*_ is the signal standard deviation and σ_*m*_ is the characteristic length scale of each dimension *m*.

We note that the SEM contrast of monolayer *h‐*BN on different iron facets can vary, making statistically relevant total *h‐*BN coverage assessment challenging. Hence, we binned *h‐*BN coverage information (see Figure 1 – red, yellow, green), and assigned values of 0 (no *h‐*BN observed, red), 0.5 (partial *h‐*BN surface coverage, yellow), and 1 (complete *h‐*BN surface coverage, green) as input parameters. *h‐*BN multilayers appear brighter with higher contrast under SEM, hence the fraction of surface covered by multilayer *h‐*BN could be assessed using contrast thresholding and used in the training set (Figure 3B). Due to the binned input parameter of *h‐*BN surface coverage, the predicted coverage parameter space is considered approximate.

The predicted parameter space shows complete h‐BN surface coverage at higher precursor temperatures, lower CVD growth temperatures, and longer CVD growth times (Figure 3C), consistent with our experimental data (Figure 1). Additionally, multilayer coverage is most strongly correlated to CVD growth temperature (Figure 3D), again consistent with our experimental data (Figure 2) and proposed growth mechanism (Figure 2J). To predict the regions in the parameter space that would result in a complete *h*‐BN film with minimal multilayer fractions, we select total *h‐*BN surface coverage to be >85%, and <20% multilayer *h‐*BN and visualize it in Figure 3E (see Figure S2, Supporting Information for surface coverage 85–100% and multilayer 10–20%) which suggests growth temperature >1000 °C and precursor temperature >80 °C regardless of growth time. Hence, we perform CVD at ≈1050 °C, ≈90 °C, for ≈120 min and observe a complete *h*‐BN film, with minimal multilayer regions that are limited to areas under wrinkles (Figure 3F).

### 2.3 Atomically Thin h‐BN Membranes for Hydrogen Isotope (H^+^/D^+^) Separations

**Figure 4:**
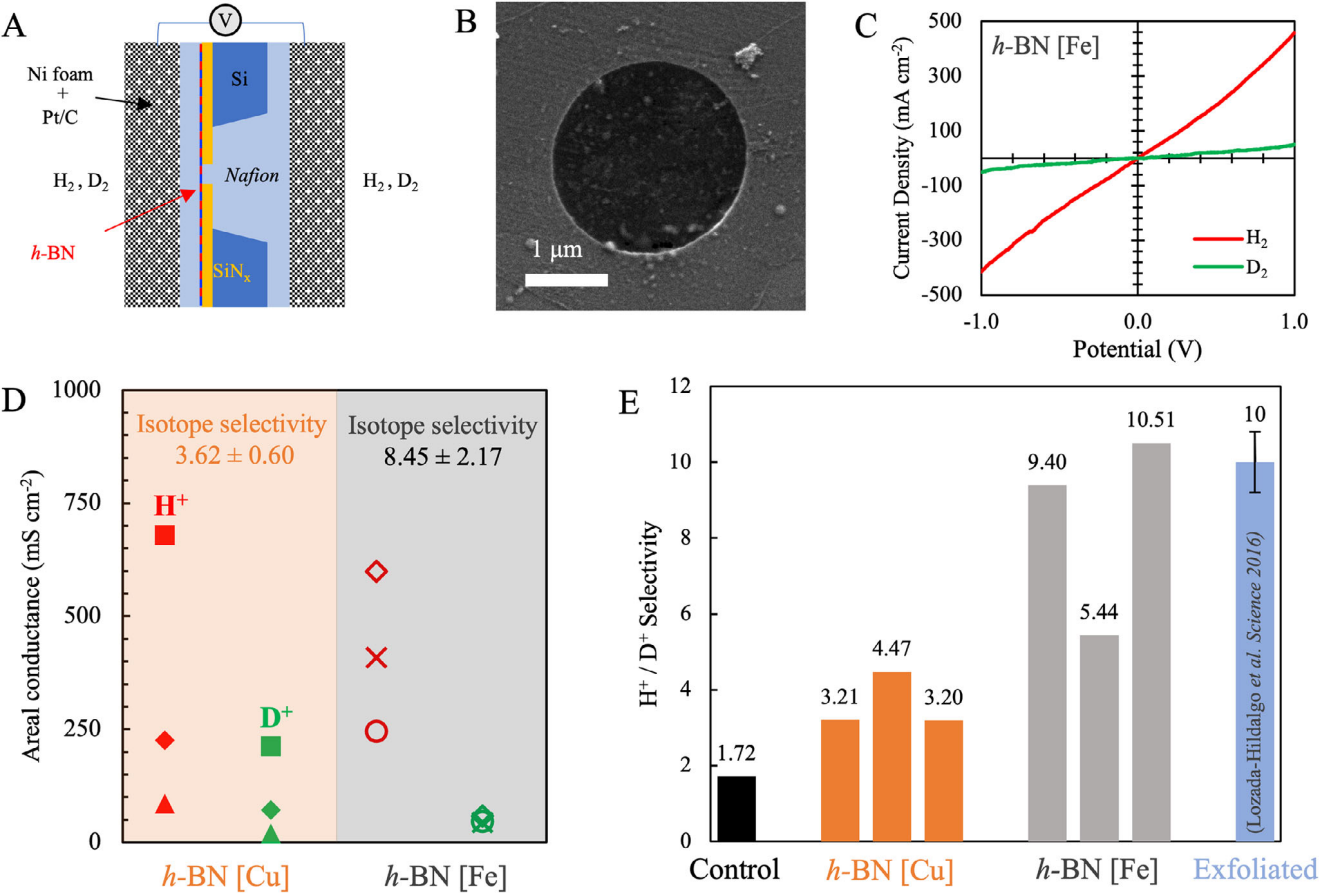
Sieving hydrogen isotopes (H^+^/D^+^) using monolayer CVD *h*‐BN membranes. A) Schematic of devices used for H^+^/D^+^ separation experiments. *h‐*BN suspended on an aperture in SiNx coated Si wafer is used to fabricate a Nafion|*h*‐BN|Nafion device. Pt/C electrodes are used to dissociate either H_2_ or D_2_ supplied at atmospheric pressure and Ni foam used to make electrical contact. Experiments are performed on the same h‐BN membranes switching between H_2_ or D_2_ feed, i.e., H_2_ gas feed humidified with H_2_O and D_2_ gas feed humidified with D_2_O. B) SEM of *h‐*BN grown on Fe foil suspended on an aperture in the SiN_x_ chip. C) Current‐voltage (I‐V) curve indicating proton and deuteron transport through *h*‐BN membranes. D) Areal conductance of protons (H^+^, red) and deuterons (D^+^, green) through h‐BN devices indicating transport rate‐based separations as well as comparison with CVD *h‐*BN grown on Cu foil ≈1050 °C. E) H^+^/D^+^ selectivity for *h‐*BN synthesized on Fe compared with h‐BN synthesized on Cu at 1050 °C. A control membrane consisting of no *h‐*BN is also shown. Notably, the h‐BN grown on Fe foil shows higher H^+^/D^+^ selectivity than *h*‐BN grown on Cu foil (also see Figures S3 and S4, Supporting Information for I‐V curves as well as Table S1, Supporting Information) and comparable to literature reports of exfoliated monolayer *h*‐BN flakes.^[^ ^3^ ^]^

To assess the quality of the optimized monolayer *h*‐BN film with minimal multilayers, we fabricate atomically thin membranes for sub‐atomic separations that leverage the difference in vibrational zero‐point energy, i.e., separation of H^+^/D*^+^* using Nafion‐h‐BN‐Nafion devices (**Figure** **4**A).^[^ ^1^, ^3^, ^4^ ^]^ Here, we transfer monolayer *h‐*BN onto Si/SiN_x_ chips containing a single ≈1–1.25 µm hole in the SiN_x_ (see Experimental Section for details), confirm *h*‐BN is suspended over the pore via SEM imaging (Figure 4B) and coat the suspended *h‐*BN with Nafion (proton‐conducting polymer) before applying nickel foam electrodes coated in a Pt/C catalyst (Figure 4A). In addition to monolayer *h‐*BN grown on Fe foil we also use monolayer *h*‐BN grown on Cu foil (see Experimental Section) since it is the most widely used catalyst for *h‐*BN synthesis via CVD.^[^ ^27^, ^56^, ^58^, ^60^, ^61^, ^62^ ^]^

Using hydrated H_2_ and deuterated D_2_ gas flow as feed, we collect I‐V curves to assess proton and deuteron transport through the membranes, respectively (Figure 4C; Figures S3, S4, and Table S1, Supporting Information). At the Pt/C electrodes, H_2_ or D_2_ is dissociated into protons or deuterons, driven through the cation‐selective Nafion polymer, through the *h*‐BN lattice and through the cation‐selective Nafion polymer on the other side wherein the H^+^ or D^+^ finally recombine on the opposite Pt/C electrode.

The areal conductance of protons and deuterons through the devices are calculated using Ohm's law, i.e., slope of the I‐V curve (Figure 4C and) and normalized to the *h*‐BN membrane area, i.e., the area of the single pore in the SiN_x_ chip. Figure 4D shows, the areal conductance of the *h*‐BN devices for D^+^ is significantly lower as compared to H^+^ and the ratio of H^+^ conductance to D^+^ conductance yields a separation factor or selectivity (Figure 4E). Notably, devices made from the optimized *h*‐BN grown on Fe exhibit H^+^/D^+^ selectivity of ≈9.4, ≈5.4, and ≈10.5 (average ≈8.45) while devices comprising of *h*‐BN grown on Cu foil at similar growth temperature ≈1050 °C exhibited H^+^/D^+^ selectivity of ≈3.2, ≈4.5, and ≈3.2 (average ≈3.62), respectively. Control devices consisting of only Nafion exhibit H^+^ conductance of ≈2.3 S cm^−2^ and D^+^ conductance of ≈1.3 S cm^−2^, resulting in a H^+^/D^+^ selectivity ≈1.7 (Figure 4E, Figure S4 and Table S1, Supporting Information for details). Pristine mechanically exfoliated h‐BN and graphene^[^ ^3^ ^]^ exhibit H^+^/D^+^ selectivity ≈10 while CVD graphene^[^ ^4^ ^]^ shows H^+^/D^+^ selectivity ≈8 indicating the presence of inherent defects can lower selectivity via non‐selective leakage.

Notably, we observed higher areal conductance for the CVD h‐BN membranes (H^+^ areal conductance ≈85 ‐ 680 mS/cm^2^), compared to values reported in literature for mechanically exfoliated h‐BN^3^ ≈100 mS/cm^2^ and note intrinsic defects (point defects, lattice vacancies, etc.) are unavoidable in CVD. However, we emphasize that *h*‐BN on Cu and Fe are grown at the similar temperature (≈1050 °C) and similar precursor flux (≈3 mg precursor heated to ≈90 °C for Fe samples, and ≈3.5 mg precursor heated to ≈85 °C for Cu samples), indicating the role of the catalyst in the quality of h‐BN synthesized as observed via a difference in H^+^/D^+^ selectivity (≈8.45 for Fe h‐BN versus ≈3.62 for Cu h‐BN). Taken together, H^+^/D^+^ selectivity ≈8.45 for h‐BN synthesized on Fe foil is indicative of high‐quality (lower defects in the *h*‐BN lattice) compared to h‐BN synthesized via CVD on Cu foil (H^+^/D^+^ selectivity ≈3.62), with values approaching those of mechanically exfoliated h‐BN ≈10 and comparable to literature reports for CVD graphene^[^ ^4^ ^]^ ≈8.

## 3 Conclusion

In conclusion, we developed a scalable Fe‐catalyzed CVD process to synthesize large‐area, high‐quality monolayer h‐BN films, overcoming key limitations of traditional ammonia‐assisted methods. By systematically investigating the parameter space—growth time, temperature, precursor delivery—and leveraging mechanistic insights, we demonstrated that increasing the growth temperature effectively suppressed multilayer formation by enhancing B and N solubility into the Fe bulk and reducing supersaturation at the surface. Machine learning‐enabled optimization of the CVD conditions allowed data‐driven identification of regimes promoting predominantly monolayer h‐BN coverage with minimal secondary nucleation or multilayer structures. Our approach achieved high quality h‐BN monolayers with uniform coverage for sub‐atomic separations, i.e., hydrogen isotope separation H⁺/D⁺ selectivity of ≈8.45 for h‐BN grown on Fe—approaching that of mechanically exfoliated monolayers and significantly outperforming Cu‐catalyzed CVD h‐BN (selectivity ≈3.62). We attribute the superior performance to the reduced defect density and higher quality of the Fe‐grown h‐BN compared to Cu‐grown films. Our study advances a cost‐effective, scalable synthesis route for high‐quality monolayer h‐BN synthesis for membrane applications and highlights the potential of machine learning‐guided CVD optimization to drive progress in the controlled growth of 2D materials for sub‐atomic separations and beyond.

## 4 Experimental Section

### h‐BN Growth

*h*‐BN growth was performed via CVD using a custom‐build reactor with a cold‐trap.^[^ ^5^, ^70^ ^]^ Briefly, ≈1 cm^2^ of Fe foil (100 µm, 99.99%, or 127 µm 99.5% Alfa Aesar) was pretreated by sonication in 0.2 M FeCl_3_ (98% purity, EDM Millepore) for 5 min, followed by rinsing and sonication in DI water to remove any surface contaminants and surface oxides. Ammonia‐borane (≈3 − 3.5 mg, ≈99% purity, Aldrich, stoichiometric B:N balance) was used as precursor for *h*‐BN CVD and loaded into a glass vial placed into the precursor chamber.^[^ ^5^, ^70^ ^]^

The cleaned Fe foil was loaded into the CVD reactor (Figure 1A) and heated in H_2_ (≈50 sccm, ≈420 mTorr) to the annealing temperature of 1050 °C at 40 °C min^−1^. After annealing for 30 min (Figure 1B) to further clean the Fe surface and facilitate reduction of the Fe surface and promote grain growth of Fe in the foil, the reactor temperature was set to the desired growth temperature 950–1050 °C while maintaining H_2_ flow. Once the reactor temperature has stabilized (≈15 min), the precursor chamber was heated to the desired temperature (≈60–120 °C, referred to as precursor temperature) with a thermal heat tape allowing precursor delivery into the reactor for the growth time (≈15–120 min) via a valve. Finally, the valve connecting the decomposition chamber from the rest of the system was closed and the CVD reactor was quench cooled to room temperature under hydrogen flow. The precursor chamber and the reactor were cleaned via a bake‐out procedure between every CVD growth run. Specifically, the reactor was wiped with isopropanol and heated to 1000 °C in air for 30 min, while the precursor chamber was heated to 120 °C for 60 min with vacuum from a roughing pump ≈3mTorr. Raman, and optical images were collected on *h*‐BN film grown on 127 µm 99.5% purity Fe foil. 100 µm, 99.99% purity Fe foil was used for all other experiments to minimize influence from impurities in the foil on *h‐*BN growth. *h‐*BN growth on Cu foil was performed at ≈1050 °C using methods reported in detail elsewhere.^[^ ^5^, ^70^ ^]^

### h‐BN Transfer

*h‐*BN transfer to 300 nm SiO_2_/Si wafer, fused quartz and SiN_x_ single aperture chips were performed using a PMMA carrier layer. Briefly, a 2 wt.% solution of PMMA in anisole was drop casted over the *h‐*BN on Fe or Cu foil and any excess solution was removed from the surface. After drying in air, the Fe/*h‐*BN/PMMA was floated PMMA side up in 0.2 M FeCl_3_ and pre‐etched for ≈20 min to remove *h‐*BN on the back‐side of the foil, before being transferred to water bath for ≈10 min and returned to the FeCl_3_ solution to completely etch the Fe foil ≈12 h. Finally, the h‐BN/PMMA stack was floated three times on DI water (≈10 min each) and scooped on the target substrate, e.g., 300 nm SiO_2_/Si wafer or fused silica wafer and allowed to dry under ambient conditions for ≈30 min. The sample was heated at ≈130 °C on a hot plate for 20 min to ensure adhesion to the substrate. PMMA was removed using acetone and the sample was rinsed in IPA before drying in air. 0.2 M ammonium persulfate (APS) was used for etching the Cu foil to transfer the *h‐*BN on Cu foil.^[^ ^5^, ^70^ ^]^

### Characterization

SEM imaging was performed on a Zeiss Merlin electron microscope ≈2 kV acceleration voltage and Image analysis via thresholding was performed using imageJ software. UV‐vis absorption spectra were collected on *h‐*BN transferred to fused quartz using a Cary 60 UV‐vis spectrometer using bare fused quartz as a baseline. A tauc plot was computed assuming a direct bandgap for monolayer *h*‐BN.^[^ ^5^, ^70^ ^]^ Optical images were acquired using an optical microscope (Amscope) after heating the *h‐*BN on Fe foil on a hotplate in the ambient environment at ≈300 °C until the un‐covered Fe foil surface color changed ≈2 min. Raman spectroscopy was performed on *h*‐BN transferred to 300 nm SiO_2_/Si wafer with a Renishaw Raman spectrometer using 532 nm excitation wavelength at Oak Ridge National Laboratory. AFM images of *h‐*BN transfer to 300 nm SiO_2_/Si wafer were obtained with a Cypher AFM system (from Asylum Research, an Oxford Instruments company) at Oak Ridge National Laboratory. Images were captured in AC mode, using commercially available Multi75‐ G (BudgetSensors) tips with a free resonance frequency of 75 kHz and a spring constant of 3 N m^−1^. Setpoint used during imaging was ≈70% of the free amplitude. STM measurements of *h‐*BN on Fe foil were obtained with an Omicron variable temperature scanning tunneling microscope (VT‐STM) at room temperature in the Center for Nanophase Materials Sciences at Oak Ridge National Laboratory. The samples were annealed under ultra‐high vacuum at 500 °C for ≈5 h before imaging.

### Machine Learning for Parameter Space Analysis and Prediction

Two gaussian process regressions were performed on experimental data sets consisting of: {growth temperature, precursor temperature, growth time, and *h*‐BN coverage} and {growth temperature, precursor temperature, growth time, and multilayer *h‐*BN coverage}.

Analysis was performed using MATLAB built‐in “fitgp” function using the “ardmatern53” kernel, corresponding to the Matérn 5/2 kernel and independent predictor scales. The independent predictor scales were found to be essential since predictor values vary over a range, i.e., growth temperature (≈950–1050 °C), AB precursor temperature (≈60‐120 °C), growth time (≈15‐120 min) and coverage (0‐1). Prediction results were plotted using MATLAB.

### Hydrogen isotope measurements

12 mm × 12 mm silicon chips with silicon nitride (SiNx) membranes with a single micropore were fabricated using photolithographic patterning and standard cleanroom processes. Fabrication started with deposition of 200 nm low stress Si‐rich SiNx on both sides of 300 µm thick double‐side polished silicon wafers using low pressure chemical vapor deposition (LPCVD). Subsequently, pores in SiNx layer on the front side of wafers were defined by optical lithography followed by anisotropic reactive ion etching (RIE) through the SiNx layer and striping remaining resist in a hot N‐methyl‐pyrrolidone. 700 nm thick positive resist (SPR955‐0.7 spun at 3000 RPM) exposed on a contact aligner was used as a masking layer. 600 µm × 600 µm windows for bulk Si etch were patterned on the back side of wafers in a similar manner. Wafers with patterned front and back side SiNx layers were etched in 30% KOH aqueous solution at 80 °C for ≈5 h. To reduce pore sizes and seal potential pinholes and defects developed in LPCVD SiNx after KOH etch, 200 to 400 nm of PECVD SiNx was deposited on the wafer with membranes facing up in the PECVD tool.

*h‐*BN was transferred from the catalytic growth substrate and suspended on an aperture (diameter ≈ 1‐1.25 µm) in SiNx coated Si wafer to fabricate a Nafion|*h*‐BN|Nafion device (see Figure 4A,B). Transfer of h‐BN from Fe or Cu growth substrate to SiNx/Si chip resulted in poor transfer, potentially due to poor adhesion between h‐BN and SiNx|Si wafer. To improve the h‐BN transfer, first CVD graphene was transferred to SiNx|Si wafer following the polymer based approach and subsequent removal of polymer, followed by a O_2_ plasma treatment (Harrick Plasma cleaner; Low power ≈7 W, ≈5 min, 500 mTorr) to completely remove the transferred graphene from the SiNx|Si aperture. Finally, h‐BN from Fe or Cu growth substrate was transferred to this chip.

To perform the ionic conductance measurements, first Nafion (5 µL of 5 wt.%) was coated on the chip (h‐BN on SiNx|Si) to provide the ionic conduction pathways for the protons (H^+^) and deuterons (D^+^) while making sure that Nafion did not spread over and contact the two sides of the chips, providing an additional ionic transport pathway (ionic sorting the device). Humidified hydrogen (in H_2_O) and deuterium (in D_2_O) work as sources for H^+^ and D^+^, respectively. A Pt/C catalysts ink (42 wt.% Nafion) coated on Ni foam was gently placed on the drop casted Nafion (before it completely dried out) and the resulting device was baked in a humidified environment at ≈120 °C for ≈30 min to improve the mechanical bonding between the electrodes and Nafion. Additionally, to further improve the mechanical stability of devices, edges of Ni foam and SiNx|Si wafer were sealed with epoxy. Next, Cu wires were used to make electrical contact with the device to make connections with a Potentiostat (Gamry). Finally, the devices were assembled inside a gas reservoir (H_2_O humidified H_2_ for H^+^ and D_2_O humidified D_2_ for D^+^) for measuring ionic conductance for protons and deuterons in a humidified environment.

Ionic measurements for H^+^ were recorded first, followed by ionic transport measurements for D^+^ by scanning the voltage from −1 to 1 V (via cyclic voltammetry, CV at a scan rate of 10 mV s^−1^) and recording corresponding current. The recorded CV plots were averaged (over two scan directions “−1 to 1V” and “1 to −1V” to minimize any capacitive/charging current) to achieve the current‐voltage (I‐V) plots. Next, before recording current‐voltage plot for D^+^ measurements, a conversion of H‐form Nafion to D‐form Nafion was achieved by measuring current (in D_2_O humidified D_2_ environment) at constant voltages of 0.5 and −0.5 V till a stable current was achieved as reported in prior literature but with an extended time of ≈1 h to ensure complete conversion from H^+^ to D^+^ form Nafion and minimal proton, H^+^ residuals.^[^ ^3^, ^4^ ^]^ Ionic conductance was extracted from the linear portion of the recorded current‐voltage plot (slope of the linear portion of I‐V plots) and isotope selectivity was computed as the ratio of ionic conductance for proton (H^+^) to deuterons (D^+^).

## Conflict of Interest

P.R.K. acknowledges stakes a company aimed at commercializing 2D materials.

## Author Contributions

P.R.K. conceived and supervised the project. P.C. fabricated devices with h‐BN membranes and performed the hydrogen isotope sieving experiments. A.E.N. synthesized *h*‐BN via CVD, performed SEM, UV‐vis absorbance and machine learning analysis. P.C. and A.N. contributed equally to this work. N.L. fabricated SiN_x_ chips at ORNL. I.V. performed Raman spectroscopy at ORNL. M.C. and L.C. performed AFM at ORNL. S.M.H. and A.L. performed STM imaging at ONRL. All authors contributed to discussions. P.R.K. wrote the manuscript using draft text from the Ph.D. thesis of A.E.N. P.C. and P.R.K. responded to reviewer comments.

## Supporting information



Supporting Information

## Data Availability

The data that support the findings of this study are available in the supplementary material of this article.
