# Supplementary material for: Sieving Hydrogen Isotopes via Machine Learning Assisted Chemical Vapor Deposition (CVD) of High‐Quality Monolayer Hexagonal Boron Nitride (h‐BN) on Iron Foils
Source: Adv Mater. 2025 Nov 28;38(7):e11868. doi: 10.1002/adma.202511868 (PMC12862682; doi:10.1002/adma.202511868)
Supplement: Supplementary file 1 — Supporting Information [file ADMA-38-e11868-s001.pdf]

ADVANCED MATERIALS

Supporting Information

for *Adv. Mater.*, DOI 10.1002/adma.202511868

Sieving Hydrogen Isotopes via Machine Learning Assisted Chemical Vapor Deposition (CVD) of High-Quality Monolayer Hexagonal Boron Nitride (h-BN) on Iron Foils

*Pavan Chaturvedi, Andrew E. Naclerio, Saban M. Hus, Ivan V. Vlassiouk, Nickolay Lavrik, Marti Checa, Liam Collins, An-Ping Li and Piran R. Kidambi**

Supporting Information:

Sieving Hydrogen Isotopes via Machine Learning Assisted Chemical Vapor Deposition (CVD) of High-Quality Monolayer Hexagonal Boron Nitride (h-BN) on Iron Foils

Pavan Chaturvedi[⊥,a] Andrew E. Naclerio[⊥,a] Saban M. Hus, Ivan V. Vlassiouk, [b] Nickolay Lavrik,[b] Marti Checa, [b] Liam Collins, [b] An-Ping Li and Piran R. Kidambi*[,c]

[a] Chemical and Biomolecular Engineering Department, Vanderbilt University, Nashville, TN
[b] Center for Nanophase Materials Sciences, Oak Ridge National Laboratory, Oak Ridge, TN
[c] Mechanical and Aerospace Engineering Department, University of Florida, Gainesville, FL
*Corresponding Author: p.kidambi@ufl.edu
[⊥] These authors contributed equally to this publication

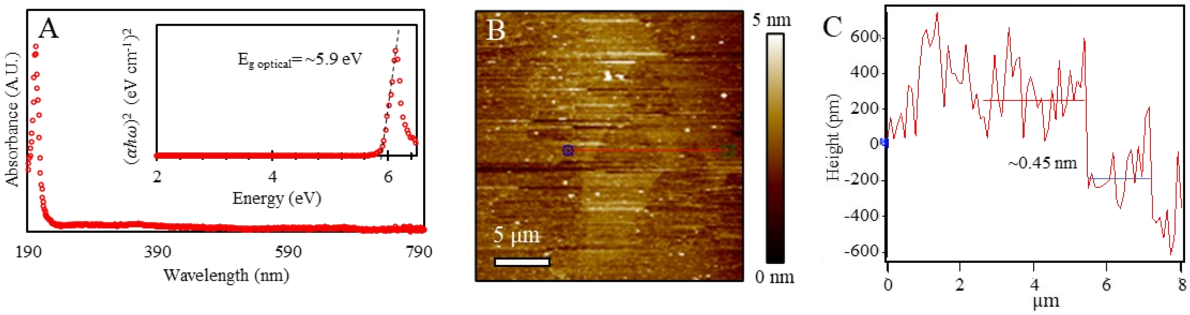

Figure S1. Characterization of monolayer h-BN synthesized via CVD on Fe foil.
A) UV-vis absorption spectra acquired on continuous *h*-BN film transferred to fused quartz. Inset shows calculated Tauc plot of *h*-BN. B) AFM image of triangular *h*-BN domains transferred to 300 nm SiO_2 /Si wafer. C) AFM height profile map for line in B.

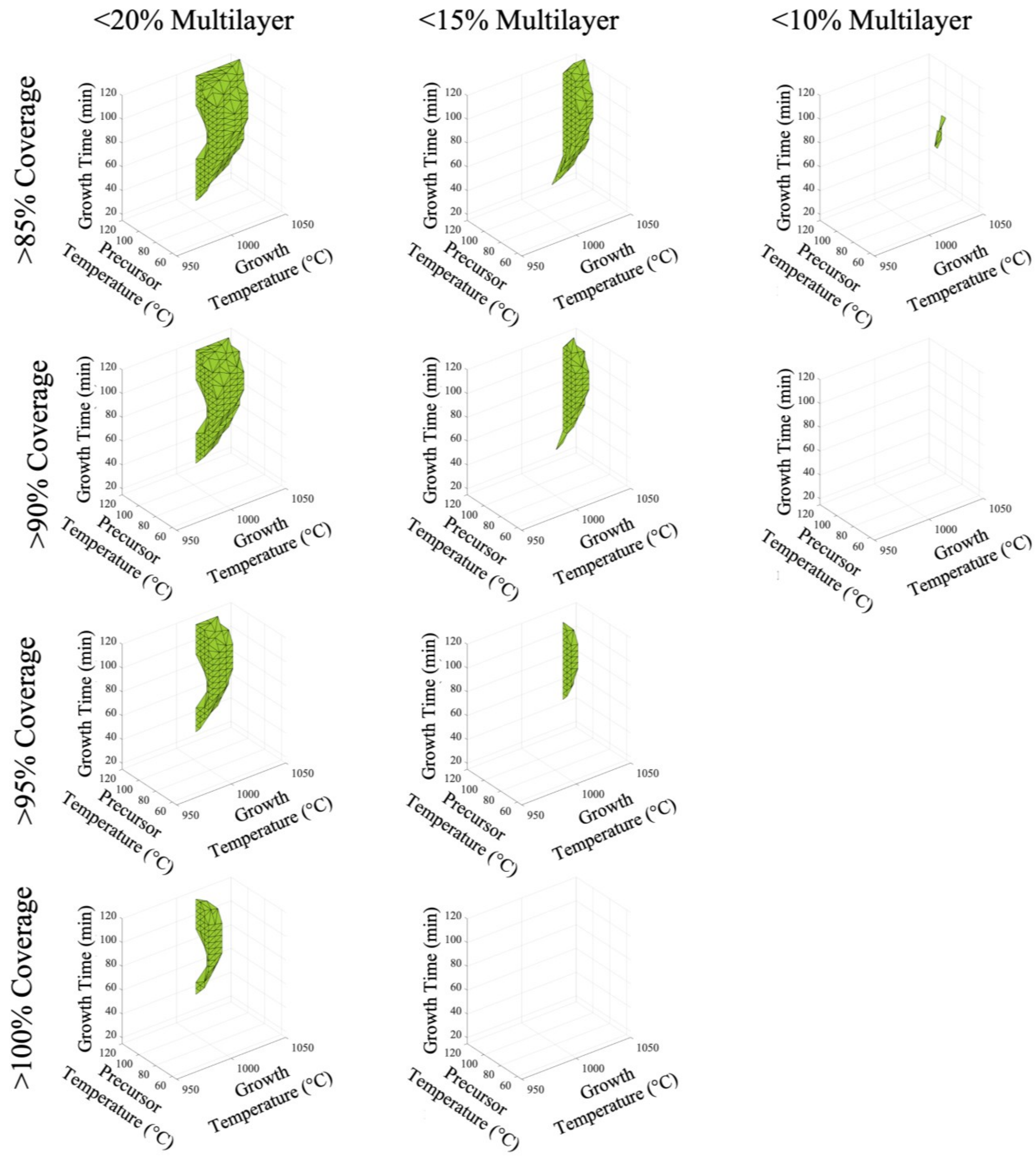

Figure S2. Machine learning model predicted parameter space for *h*-BN CVD on Fe foil.
Parameter space computed for *h*-BN surface coverage of > 85% - 100%, and *h*-BN multilayer coverage <20 – 10 % as a function of growth temperature, precursor temperature, and growth time.

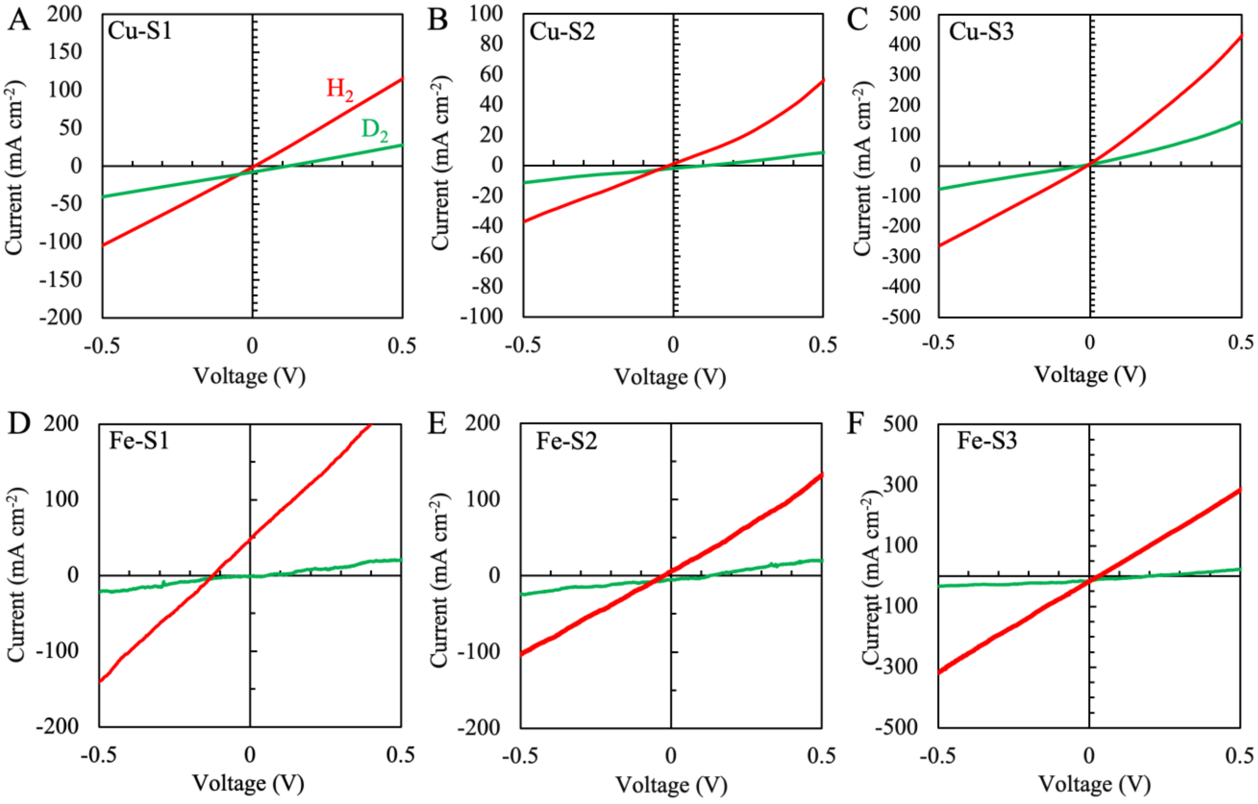

Figure S3. I-V curves for *h*-BN devices.
A-C) I-V curves of devices containing monolayer *h*-BN grown on Cu foil at 1050 °C for H_2 (red) gas feed humidified with H_2O and D_2 (green) gas feed humidified with D_2O. D-F) I-V curves of devices containing monolayer *h*-BN grown on Fe foil at 1050 °C for H_2 (red) and D_2 (green) gas feed. Also, see Figure 4.

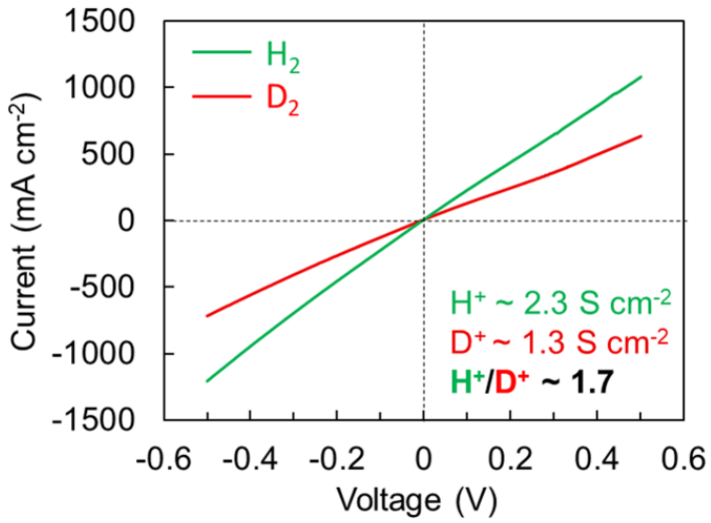

Figure S4: I-V curves for control device without h-BN.
I-V curves for SiNx chip with aperture + Nafion but without h-BN (control device) for H_2 (red) gas feed humidified with H_2O and D_2 (green) gas feed humidified with D_2O.

Table S1. Resistance values extracted from I-V curves in Figure S3 and S4.

Sample	Areal conductance (mS cm^{-2})		Areal resistance (Ω cm^2)		Selectivity
	H^+	D^+	H^+	D^+	
Cu h-BN S1	679.0	211.8	1.5	4.7	3.2
Cu h-BN S2	85.0	19.0	11.8	52.6	4.5
Cu h-BN S3	225.0	70.4	4.4	14.2	3.2
Fe h-BN S1	408.0	43.4	2.5	23.0	9.4
Fe h-BN S2	245.0	45.0	4.1	22.2	5.4
Fe h-BN S3	599.0	57.0	1.7	17.5	10.5
Control w/o h-BN	2267.0	1300.0	0.4	0.8	1.7
